# Analysing Touchscreen Gestures: A Study Based on Individuals with Down Syndrome Centred on Design for All

**DOI:** 10.3390/s21041328

**Published:** 2021-02-13

**Authors:** Jorge Martin-Gutierrez, Marta Sylvia Del Rio Guerra

**Affiliations:** 1 Department of Techniques and Projects in Engineering and Architecture, Universidad de La Laguna, Av. Angel Guimerá sn, 38071 Tenerife, Spain; 2 Department of Computer Science, Universidad de Monterrey, Av. Ignacio Morones Prieto 4500-Pte., San Pedro Garza García, 66238 Nuevo Leon, Mexico

**Keywords:** hand gestures, human computer interaction (HCI), user-centered design, Down syndrome, user experience, UX guidelines

## Abstract

There has been a conscious shift towards developing increasingly inclusive applications. However, despite this fact, most research has focused on supporting those with visual or hearing impairments and less attention has been paid to cognitive impairments. The purpose of this study is to analyse touch gestures used for touchscreens and identify which gestures are suitable for individuals living with Down syndrome (DS) or other forms of physical or cognitive impairments. With this information, app developers can satisfy Design for All (DfA) requirements by selecting adequate gestures from existing lists of gesture sets. Twenty touch gestures were defined for this study and a sample group containing eighteen individuals with Down syndrome was used. A tool was developed to measure the performance of touch gestures and participants were asked to perform simple tasks that involved the repeated use of these twenty gestures. Three variables are analysed to establish whether they influence the success rates or completion times of gestures, as they could have a collateral effect on the skill with which gestures are performed. These variables are *Gender, Type of Down syndrome,* and *Socioeconomic Status*. Analysis reveals that significant difference is present when a pairwise comparison is performed, meaning individuals with DS cannot perform all gestures with the same ease. The variables *Gender* and *Socioeconomic Status* do not influence success rates or completion times, but *Type of DS* does.

## 1. Introduction

It has become much more commonplace to find touchscreen devices— tablets, smartphones or e-readers, amongst others—and touch gestures being used in both personal and professional settings. This situation has led to a change in people’s habits [1,2]. The natural and intuitive interaction [3] between the end-user and touchscreens via touch gestures has transformed smartphones and tablets into the most widely used pieces of technology in today’s society [4]. In fact, interactive screens are so natural and intuitive that studies such as that by Plowman confirm that even small children are capable of using these devices, and do so even before verbal communication development is completed [5]. In the report by Media and Redeout [6] on the use of technologies by children aged under 8 years old, the authors indicate that 38% of participants knew how to use a smartphone, iPad, or similar piece of technology, and that 10% of these participants were aged between 0–23 months old and 39% aged between 2–4 years old. According to the study carried out by Hourcade et al. [7], it’s possible to affirm that these percentages are higher today because these sorts of devices are used at increasingly younger ages. Even adults use them when they are placed in their surroundings, i.e., urban interfaces such as parking meters, on-street cycle parking, cash machines, ticket machines at the underground station, etc.

Mobile devices and apps are being used more and more frequently to help resolve work related tasks, making the end-user more productive and efficient [8,9,10]. There is no doubt that for many people their smartphone or tablet has become an indispensible tool in the workplace. However, in the private setting touchscreen devices are gradually substituting other devices in an attempt to make users’ lives easier and less cluttered, which explains why society is becoming increasingly dependant on such devices [11].

Natural and intuitive multi-touch systems are based on touch and direct manipulation. Shneiderman and collaborators put forward three ideas for the concept of direct manipulation: (1) the visibility of objects and actions of interest; (2) the replacement of typed commands with pointing-actions focused on objects of interest; (3) rapid, reversible and incremental actions that help to control the technology whilst avoiding complex instructions [12]. Hourcade, on the other hand, argues that direct touch on the screen is preferable to using a mouse when selecting options offered onscreen [13].

Ingram et al. indicate that the number and types of multi-touch interactions that end-users and app developers “have instinctively agreed upon” is restricted [14]; in other words, the interactions needed in order to use an app are generally limited to the following: a single touch in order to select something; a single finger to move something; two fingers to scale something; and, to a lesser extent, two fingers to rotate something. Thus, the lack of consensus regarding standardized interaction, and the fact that there are already some universally accepted gestures, makes the need for well-designed multi-touch interactions absolutely crucial.

In order to design touchscreen interfaces that are better suited to end-user needs, Jeong & Liu studied how humans use their fingers whilst using touchscreen devices, and examined the different characteristics of gestures that are used on the touchscreen (gesture performance, time, error rate, speed, and finger movement trajectory) [15].

It is true that user experience (UX) is taken into account when apps or websites are designed in order to adapt them to the user needs. Therefore, it is possible to encounter apps for children living with Down syndrome or autism [16], or for the elderly [17]. However despite this being the case, during the design process little to no consideration has been paid to touch gestures to ensure that screen interactions have been designed to suit all users. Usability studies have been performed for multi-touch screens aimed at different collectives: children [18,19], adults and the elderly [20,21,22], and individuals living with Down syndrome [23,24], amongst others.

According to the statistics portal Statista [25], the app market has grown substantially in recent years. In 2018, figures for app downloads worldwide reached 205.4 billion and it is estimated that this figure will grow to reach 258.2 billion by 2022. The most frequently downloaded apps (not games) in Google Play Store in 2018 were Facebook, WhatsApp and Google, although mobile games were also downloaded in large volumes on mobile devices. In light of this set of circumstances pertaining to the use of and dependence on technologies that rely on touch gestures, it should be noted that individuals living with disability run the risk of being sidelined and are at the risk of social exclusion. This would increase the digital divide and, consequently, social inequality [26]. 

Some authors state that the digital divide is a gap that exists between those using advanced technologies as part of their day-to-day lives and those who encounter difficulties in using such technologies [27]; these difficulties may emerge as the result of being unfamiliar with such technology, living with some form of disability that makes handling such technology difficult, holding the perception that such technology is too complex, or simply because it has not been designed with all types of users in mind and cannot be manipulated by all users. The digital divide is transforming into an element that separates and excludes people, collectives and countries [28].

Usually the literature uses the term "Design for All" to refer to developing applications and products that are accessible to everyone. “Design for Inclusion” encompasses three main design principles: Design for All (DfA), Inclusive Design (ID) and Universal Design (UD). These were created to address diversity in age, gender, culture, abilities, disabilities and technological literacy. Design for Inclusion is based on the assumption that most users are not the standard-user [29]. Thus, one of the most ignored disabilities is cognitive impairment. DS users must be included in a Design for Inclusion approach.

This article focuses on individuals living with Down syndrome (DS) as users of touchscreen devices that require the use of gestures for interaction purposes.

Despite UX conditions having been taken into account when designing applications [30,31] and usability studies performed to improve said design [32,33], the fact remains that certain physical gestures used by end-users (touchscreen—Kinect gestures—Mid-Air gestures) that are then interpreted by devices (screen, Kinect, VR/AR goggles, etc.) influence the interactive capacity between the user and machine. What is more, they have not been studied sufficiently to ensure they address the physical limitations experienced by certain collectives [34].

Individuals with DS have varying degrees of cognitive delay [35], which is reflected in lower IQ scores when compared against the general populace [36]. This indicates the presence of mental impairment [37] and it has been established that this impairment worsens with age [38]. Individuals living with DS find themselves affected by three main types of impairment: cognitive, motor, and perceptual [39]. These affect their gross and fine motor skills, cognitive perception, speech perception, sensorial perception and processing, and communication skills [40,41]. Current designs of mobile devices and touchscreens utilise sets of established gestures. Individuals with DS, however, possess unique physical and cognitive traits that make it difficult for them to perform certain touch gestures. As such, a developer’s design choices and gesture selections may hinder the user experience of current devices by individuals with DS. The driving motivation behind granting individuals with disabilities full access to ICTs is to ensure they do not suffer from another form of social exclusion and that their rights are protected; these are technologies that are used in the personal, professional and education settings, and thus equal access to these technologies guarantees equal access to the breadth of educational and professional opportunities on offer. Furthermore, these technologies provide these individuals with alternative ways to socialize.

The aim of this work is to establish the most suitable touchscreen gestures for individuals with DS, and ensure they are considered as part of UX when designing inclusive apps. From a social inclusion standpoint, this study aims to assist developers in developing apps that use interactive gestures that can be performed by all users. Several contributions to the literature are made by this work: firstly, the development of an app that is capable of measuring gestures and detecting new types of interactions; secondly, findings from an experimental study on all types of possible interaction involving finger and hand gestures by individuals with DS of all ages, socioeconomic status and gender; and finally, a set of design guidelines that should be taken account when designing touchscreen apps. 

## 2. Related Works

Recently Nacher introduced a work where measured touchscreen interactive gestures by children with DS [24]. From their research, it should be noted that the motor impairments experienced by individuals living with DS worsen with age as Barnhart and Connolly, already pointed out [42]. The results presented in the mentioned Nacher work, indicate that the seven gestures studied are successfully executed by children with DS. However, the work presented in this paper, unlike the aforementioned, includes a much broader range of gestures. A total of 20 gestures are analysed, including gestures performed with the fingers of one hand, the fingers of two hands, a single hand, and both hands.

### 2.1. Interaction and Gestures

In recent decades the development of touchscreen technologies has proliferated and there have been many studies on the use of touchscreen devices and gestures in order to better understand users’ needs usuarios [43,44,45,46,47,48]. Although conventional touchscreen technologies only allowed for the use of a limited number of finger gestures, e.g., writing and selecting objects (normally with the index finger), the range of touchscreen gestures and the fingers used has since grown in recent years. For example, Mayer, propose using the positioning of fingers to enrich input [49]. Crescenzi & Grané found that young children adapted their gestures to the app content, which meant more natural gestures were used than those originally designed by the app developer [50]. It should be noted the classification of gestures based on their taxonomy category (nature, flow, dimension, complexity, interaction and location) Dingler and collaborators who also proposed a methodology for designing ‘*consistent gestures across different devices*’ [51].

Touchscreen gestures that use fingers generally fall under two categories: single-touch gestures and multi-touch gestures. Single-touch gestures include clicking (or pressing, holding) an object, or sliding (or moving, dragging) an object. However, multi-touch gestures include pinching (or zooming in) and stretching (or zooming out).

The multi-touch gesture dictionary and work of Plichta et al. describes a plethora of entries for interacting with desktop computers, tablet computers, notebook computers, handheld computers, media players, mobile phones, and so on [52,53].

According to Lim et al. the research on gesture interaction focuses on two facets: on the one hand it analyses gesture recognition within systems; and on the other hand it analyses usability factors, studying design and natural and intuitive gesture communication that takes physical and cognitive traits into consideration [54]. On the other hand, to evaluate the ease of use of gestures, Jeong and Liu analysed the speed at which a series of gestures were executed [15].

### 2.2. Interaction and Individuals Living with Down Syndrome

The main objective of usability is to include intuitive design for all users, including users living with DS. Unfortunately, conventional research methods do not contemplate all the needs of this group of people [55].

Recent studies show how technology has been used with individuals living with DS, whether it be to assist learning, or performing tests that measure the usability of apps and systems so that they are accessible to users, i.e., BeeSmart and Eye-hand Coordination of Children with Down Syndrome [56]. Users with DS are able to use computers because they can use devices such as the mouse, keyboard and screen [57]. In particular, a lot of research has been performed with this social group using games [58,59,60]. 

When reviewing the bibliographic summary performed by Cáliz and collaborators, it was possible to identify 98 scientific contributions for usability studies with mobile devices [55]. Out of these, only five had been performed taking into account individuals with DS: Nacher and collaborators studied interaction on touchscreen devices, but only reviewed four gestures [24]; and Mendoza and his collaborators analysed how the eight most commonly used touchscreen gestures are learnt by teenagers with DS [61]. Thus, the authors of this paper felt it necessary to broaden the scope of such research, believing it should include more gestures and focus on gesture use in order to create applications under the paradigm of user-centric design.

## 3. Problem Statement 

Down syndrome is a genetic condition caused by a third copy of chromosome 21, meiotic or mitotic nondisjunction, or an unbalanced translocation of said chromosome pair [62]. During reproduction both parents pass their genes on to their children, which are carried in chromosomes. When the baby’s cells develop, each cell is supposed to receive 23 pairs of chromosomes to ensure the correct total of 46 chromosomes; half of these chromosomes are from the mother and half are from the father. However, in the cells of an individual with DS, there is an extra pair of chromosome 21, meaning that instead of having 46 chromosomes they have 47. This extra chromosome has a direct effect on both the brain and body’s physical development and has been identified as the cause of the unique physical and cognitive traits found in DS.

One of the predominant traits found in DS is limited motor movement, which is a consequence of delayed motor development [63]. Kim and collaborators, affirm that at a young age children with DS display the same development as other children, however the delay in motor development becomes increasingly apparent as individuals age and remains present throughout all later stages of development. Other studies that have been performed have also made the same observation [64].

The particular physical and cognitive traits of people with Down syndrome affect individual capabilities. Persons with DS typically share a host of recognizable physical traits that include: shorter extremities; stubby fingers; a curved little finger—known clinically as Clinodactyly; a short thumb that is more widely separated from the other fingers; and, a third finger that is typically longer than the rest [65]. Another trait that tends to be encountered is microcephaly, or in other words, reduced diameter of the head and flattened occipital bone. Regarding sight, individuals with DS will often have strabismus, which is almost always a convergent squint [66]. These physical traits might negatively affect the use of electronic devices of domestic use, as they might not have been designed for inclusion. On the cognitive aspect, it is common for people to have reasoning and learning problems, but it is usually mild or moderate. They have very short attention spans, lack of logical thinking, impulsive behavior, slow learning and delay in language and speech development [67]. Troncoso and del Cerro, report all traits of persons with DS [68].

As briefly mentioned above, from a medical stance there are three types of DS [69]. All three types results from chromosome disorders affecting chromosome pair 21:

*Trisomy 21*: Accounts for 95% of cases—this is not an inherited condition and happens by chance. It occurs when all cells contain an extra chromosome. This is the most widespread type of DS.

*Translocation*: Accounts for approximately 4% of cases—this occurs when an extra fragment of chromosome 21 adheres to another chromosome.

*Mosaicism*: Accounts for 1% of cases—this occurs after the initial cell division process takes place. The nondisjunction of chromosome 21 affects some but not all cells, thus causing some cells to contain the normal number of chromosomes (46), whilst others contain an extra chromosome (47). In other words, some cells have Trisomy 21, and others do not. 

According to the National Down Syndrome Society (NDSS), about 1 in 700 infants in the United States is born with DS. It’s the most common genetic disorder in the United States to affect all social classes. As individuals with DS are able to manipulate devices such as the mouse, keyboard and computer screen, end-users with DS do use computers [36,57]. In these usability studies with individuals with DS that related to work tasks, it was found that when using a keyboard users would type more slowly because they only used one of the fingers on one of their hands. They also studied the usability of apps on touchscreen devices and the conclusion draw was that simple gestures are, in theory, easier for users with DS due to them only using one finger [70]. It has been demonstrated that the challenges that individuals with DS encounter when using machinery arise as a direct result of the physical and cognitive limitations associated with the condition.

### 3.1. Objectives and Hypothesis

The main objective of this work is to investigate the suitability of a set of touchscreen gestures for individuals with DS that use finger gestures and hand gestures. Following analysis the authors shall establish which of these gestures that can be successfully completed by individuals displaying cognitive delays associated with DS, and, from a UX stance, identify those gestures that are best suited to the widest possible audience. The gestures shall be written into Best Practice so they are taken into account when designing touchscreen devices or apps for touchscreens.

Following the software metrics approach "goal, question, metric" (GQM) [71], it is possible to state that the purpose of this study is to analyse and propose touch gestures that are suitable for individuals with DS to use when interacting with touchscreens.

In this study the authors contemplate the influence of three variables on the success rate and completion time of gestures: *gender, type of Down syndrome,* and *socioeconomic status*. The latter is under consideration as a consequence of other studies indicating that differences in intellectual abilities exist in relation to this variable [72], and that it may indirectly influence the dexterity with which gestures are performed. The hypotheses established for this piece of research are as follows:

**First Research Hypothesis** **(HR1):** *Touchscreen gestures pose different levels of difficulty for individuals living with Down syndrome.*

Gestures are determined to be a ‘success’ or ‘fail’, which is to say different gestures present different degrees of difficulty.

**The Null Hypothesis** **(Ho_1_):** *The gestures analysed have the same degree of difficulty.*

**Second Research Hypothesis** **(HR2):** *The gesture used has an effect on completion time.*

A relationship has been established between time and the successful completion of a gesture; in other words, the authors aim to establish whether the degree of difficulty of a gesture affects the time it takes to execute the gesture.

**The Null Hypothesis** **(Ho_2_):** *Gesture difficulty is related to completion time.*

**Third Research Hypothesis** **(HR3):** *Gender has an effect on the success rate of a gesture*.

**The Null Hypothesis** **(Ho_3_):** *The gender of a person with DS does not influence the success rate of a gesture.*

**Fourth Research Hypothesis** **(HR4):** *Gender has an effect on the completion time of a gesture.*

**The Null Hypothesis** **(Ho_4_):** *The gender of a person with DS does not influence the completion time of a gesture.*

**Fifth Research Hypothesis** **(HR5):** *The type of Down syndrome has an effect on the success rate of a gesture.*

**The Null Hypothesis** **(Ho_5_):** *The type of Down syndrome influences the success rate of a gesture.*

**Sixth Research Hypothesis** **(HR6):** *Socioeconomic status has an effect on the success rate of a gesture.*

**The Null Hypothesis** **(Ho_6_):** *Socioeconomic status does not influence the success rate of a gesture.*

**Seventh Research Hypothesis** **(HR7):** *The type of Down syndrome has an effect on the completion time of a gesture.*

**The Null Hypothesis** **(Ho_7_):** *The completion time of a gesture is not influenced by the type of Down syndrome.*

**Eighth Research Hypothesis** **(HR8):** *Socioeconomic status has an effect on the completion time of a gesture.*

**The Null Hypothesis** **(Ho_8_):** *Socioeconomic status influences gesture completion time.*

## 4. Experiment Design

To test the hypotheses presented, the authors of this paper designed a research study that consisted of developing an application capable of registering all gestures performed by users whilst completing tasks inside the app. To do so, a study had to be performed of the large number of previously existing apps that require touchscreen gestures in order to identify the number and type of gestures that would need to be evaluated. Next a purpose-built gaming app was created that would elicit these gestures. The app includes several different tasks that are performed by users using interactive touchscreen gestures. The app logs the personal details of users, and for future analysis it also records gesture completion times, success rates, and the size of the object the user is interacting with onscreen [73]. Once the purpose-built app was ready, the authors contacted centres that support individuals with DS in order to recruit volunteers for the study, ensuring that different social classes and different types of DS were represented in the sample group.

### 4.1. Participants

For the purposes of this research project, participant recruitment was limited to individuals with DS living in the metropolitan area of Monterrey (Mexico). Prior to commencing the study, a statistical review was performed to determine the appropriate sample size needed to guarantee reliable results.

Given the fact that the research project was going to cover the metropolitan area of Monterrey, the authors began by establishing the number of residents in the area according to the National Institute of Statistics and Geography (INEGI). This figure stood at approximately 4,225,000 individuals in 2017. Data from the same institute indicate that the number of births with DS in the country of Mexico stands at 3.73 out of every 10,000 births, whilst in the state of Nuevo Leon it stands at 1.587 out of every 10,000 births [74]. Having established the number of inhabitants, the percentage of individuals with DS, a statistical confidence level of 95%, a margin of error of 20%, and heterogeneity of 50%, it was then possible to establish that the minimun sample size for the research project is 18 participants. Despite qualitative studies indicating that just six to nine participants [75] are required in order to identify usability problems in an application or product during Think Aloud testing, more recent studies [76] suggest 10 ± 2 participants are in fact required. The research project was performed with the sample size of 18 individuals with DS aged between 9 and 34 years old (mean M = 21.83, standard deviation SD = 5.85). The sample consisted of 13 men and five women. Thus, by gender fewer women participated than men, making up only 27% of the sample size. Participants were drawn from three centres that offer support to individuals with DS. The centers were chosen from the socio-economical population who they serve. Two are private institutions, and the third one is a public one located in an underprivileged neighborhood, whose users have very limited resources. The second one, located in a middle-class neighborhood, is financed by donations and a fee payed by users. The third center (PISYE Center at Universidad de Monterrey), is a private one, and users are required to pay a very expensive tuition to attend.

The selection of those institutions was done as a means to ensure that persons of each socioeconomic status were included in the sample (High 28%, Medium 50% and Low 22%). All participants of High and Medium socioeconomic status were already users of mobile devices. Only 2 participants of Low socioeconomic status had never used a tablet.

To avoid skewing the tests, the researchers made themselves available to participants as and when participants and centres were available. Participants’ ages proved not to be an important parameter in the research project, whereas the type of DS that participants had (Trisomy 21, Translocation or Mosaicism) did prove to be an important parameter (see Table 1).

### 4.2. Gesture Selection and Description of Tasks

As the authors of this paper had little experience with DS users, empirical observations were used first to determine the type of apps these users preferred, and how often they used a smartphone. Results varied greatly, with some users proving themselves extremely adept at using a smartphone. In the light of these observations, it was decided that similar tasks as those performed in videogames would provide the best tool for measuring gesture use. It was decided to program an app that included small tasks to perform gestures on a touch screen. The app would record data such as success rate and time of completion. Next, a list of the main actions used in touch gestures was created, and a purpose-built task devised to elicit each gesture. The tasks are described in greater detail in Table 2, Table 3, Table 4 and Table 5.

### 4.3. Equipment and Software

**Figure 1:**
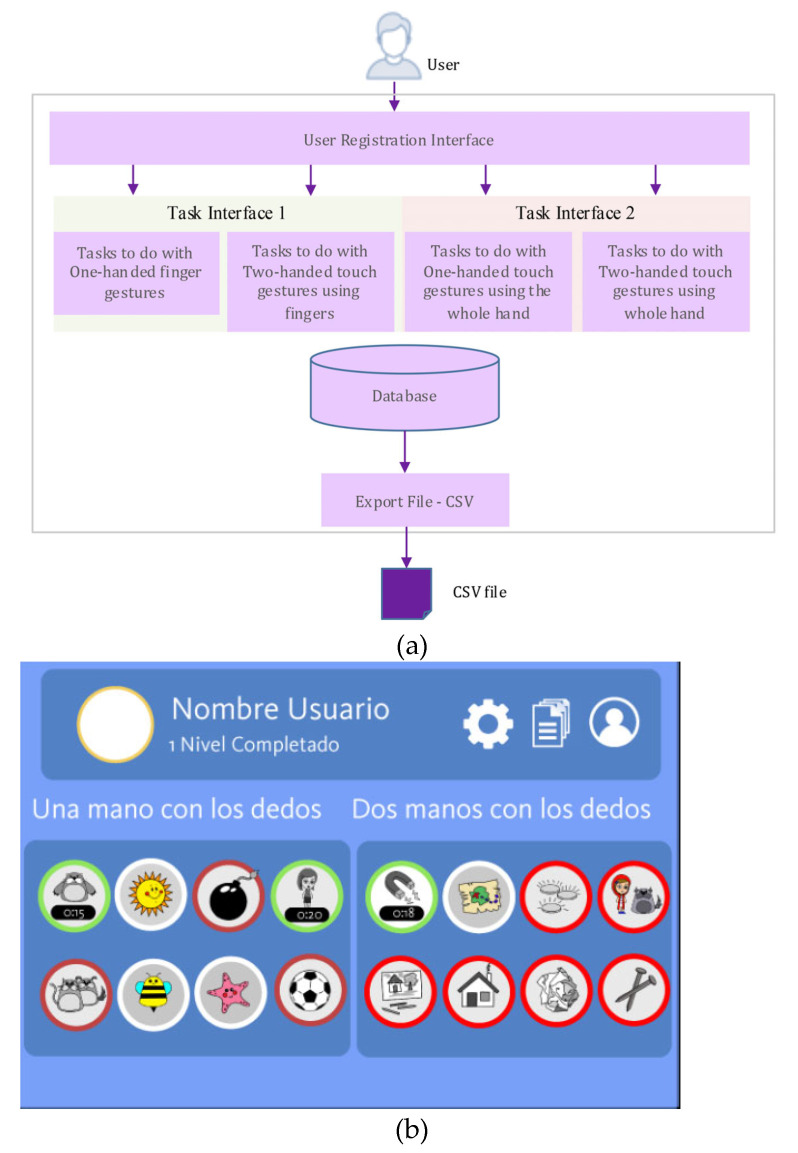
(**a**) Architecture of DS-Touch Software. (**b**) Interface software.

The purpose of the developed app, referred to as DS-Touch, was to register data from gestures performed on the touch screen. DS-Touch can record the participant’s personal information, and all gestures the person performs. The interaction framework for the experiment was implemented using the iPad’s native Xcode and C# in Visual Studio 2015. It is possible to access tasks grouped in four categories: gestures using the fingers of one hand, gestures using the fingers of both hands, gestures using the whole hand, and gestures using both hands. All information is stored in a SQLite database and can be exported to a CSV file as, and when, it is required. Figure 1a describes the software architecture of the DS-Touch app and Figure 1b displays the menu of the software interface. 

The app recognizes a gesture as successful, when the target is reached, and it is considered a failure when a predetermined amount of time elapses without the target being reached. Once the task is completed, it is repeated randomly. The data collected include user identification, success rate, and completion time. The hardware used during the execution experience was an iPad3 tablet, with a resolution of 2048×1536, 3.1 million pixels at 264 ppi, multi-touch screen, an A5X Processor, quad-core graphics and 1 GB of RAM. 

### 4.4. Procedure and Measurements

**Figure 2:**
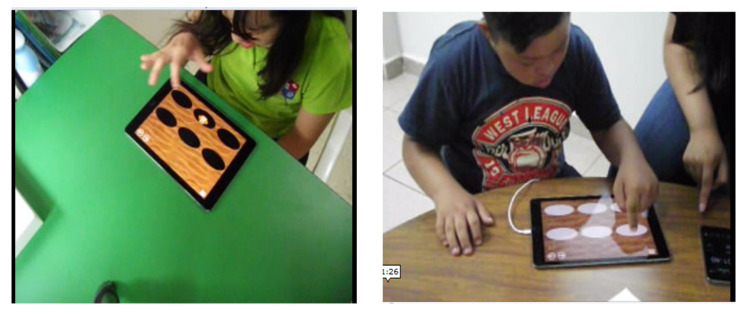
Participants (**left** and **right**) interacting with touchscreen (notice moderator showing participants how to perform gesture and finger movements).

The study was performed in three centres that offer support to individuals living with DS: the Centro Integral Down, A.C., the Down Institute of Monterrey managed by Down association of Monterrey, and the DIF of Santa Catarina. Participants were selected by the Centre’s based on their availability to avoid the risk of skewed results. Prior to the experiment all the participants, family and directors of centres were given an informed consent form reviewed by the university’s institutional review board, and provided with a brief introduction of the purposes and nature of the study. All participants gave their informed consent to be included in the study before participating. The study was conducted in accordance with the Declaration of Helsinki. The study was run over a two-day period for 5 h per day. During this time, researchers worked with 3–4 participants around one hour and half per day. The study was supervised at all times in each Centre by one of their in-house psychotherapists. The study participants were informed that they would be “playing games” using an electronic tablet. A moderator was on hand to assist participants, despite the fact that they were given verbal instructions prior to commencing (Figure 2). For ease of use, the device was placed on a table at all times and so participants could use both hands to perform the gestures. Each task was presented in a loop, so gestures were repeated in a random length of time. For each attempt, we registered the completion time and success rate. 

The moderator could change the time frame if he thought the gesture was completed successfully for a number of times or if he deemed the participant could not perform the gesture after repeatedly trying to do so. In either case, participants were to essay the next gesture. 

In more than half of the cases the moderator had to show the participants how to perform each of the gestures in order for them to complete the set task. A total of twenty gestures classified into four categories were analysed: those using a single finger on one hand, those using two fingers on one hand, those using a whole hand, those using two hands. Despite a brief set of instructions being displayed onscreen, participants also needed to receive verbal instructions as the majority of users had poor reading comprehension skills, or no reading comprehension skills whatsoever.

During each task the purpose-built application recorded: the number of attempts, the number of successfully completed gestures, and the time taken to complete a gesture; and for incomplete gestures the app measures the percentage by which they were completed. Whenever a gesture was successfully completed an audible sound was produced to provide users with feedback on their performance. Users were given five seconds to complete a task, but generally gestures would be completed in under five seconds. Once all the data were collected they were analysed to determine whether there were any differences in the success rates between different gestures. In other words, researchers attempted to establish whether certain gestures were more suited to individuals with DS than others.

## 5. Results

To test the hypotheses set forth in Section 3.1, the data gathered for gestures have been analysed in different steps: the first step consists of comparing the success rates against failure rates for each type of gesture; next, the success rate for each gesture is compared against the variables of *gender, socioeconomic status,* and *type of DS*; the third step consists of comparing the completion time for each gesture against the three aforementioned variables; and then data analysis concludes with observations made during the study and other qualitative results.

### 5.1. Comparing Gestures 

The statistical test *Pearson’s Chi-square test* (χ²) is used to determine whether the gestures have the same degree of difficulty. The Chi-square test allows us to determine the relationship between two categorical variables, thereby analysing the relationship between two qualitative variables (type of gesture and success rate): on one hand, the twenty (20) gestures described in Section 4.3, and on the other hand, the execution of the gesture with two possible outcomes (success or fail).

**Figure 3:**
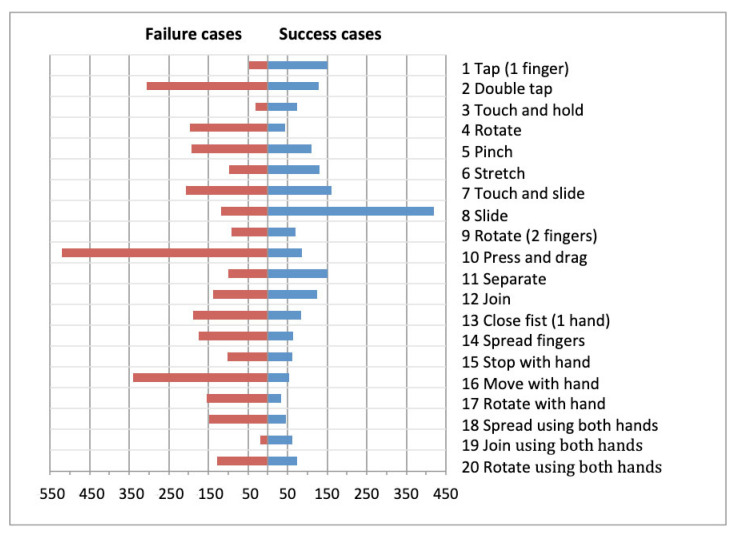
Success and failure cases by gesture.

Once the comparison of all gestures has been completed the resulting Pearson’s Chi-squared value stands at χ² = 1042.863 and the *p*-value = 0.000, indicating that there is significant different between the gestures. In other words, from a statistical stance there is significant difference between success rate and type of gesture. As such, research hypothesis HR1 is *accepted*. This analysis indicates that for individuals with DS the execution of different gestures on a touchscreen entails different degrees of difficulty, depending on the gesture in question. Furthermore, in Figure 3 it is possible to see that in the majority of instances the failure rate for gestures is higher than the success rate: out of a total of 5420 gestures that were analysed, 3293 were not completed correctly whilst 2127 were completed correctly (60.8% *failed attempts* against 39.2% *successful attempts*). It should be noted that some gestures are classified as ‘fails’ either because they were not performed correctly or because they were not fully completed. In other words, the execution of the gesture (e.g., Tap or Double tap), as well as the degree of execution of a gesture (e.g., Touch and slide) determine whether it is deemed to be a ‘success’ or ‘failure’. In the latter case, users have been observed correctly beginning and successfully executing a certain percentage of a gesture, yet failing to complete the entire gesture.

In light of the observed data, a priori it is possible to state that success rates are higher for the following gestures: Tap; Touch and hold; Stretch; Slide; and Separate. Likewise, it is possible to state that failure rates are higher for the following gestures: Press and drag; Double tap; Rotate using 2 fingers (both on same hand); Rotate using 2 fingers (one on each hand); Rotate with one hand; Pinch; and Move with hand.

Researchers ran a deeper analysis by performing a pair-wise comparison of gestures to test whether success rates were independent of gestures. This analysis was performed for each group of gestures (fingers-one hand, fingers-two hands, one whole hand, two whole hands). Once again, Pearson’s Chi-Square test was used with a significance level of *p* < 0.05. A Bonferroni correction was performed, thus establishing the levels of significance as a consequence of applying a large number of comparisons (according to group of gestures: 28, 6, 10 or 3 hypotheses). Results of the statistical analysis are displayed in Table 6, Table 7, Table 8 and Table 9. Each cell contains the *p*-value significance level for each of the gestures (shaded cells display analysis of success rate; un-shaded cells display analysis of completion time).

These tables reveal that not all gestures have the same success rate, and as such, in relation to the statement expounded in research hypothesis HR1, it is possible to once again confirm *“Touch gestures have a different degree of difficulty for individuals with DS”.* It is evident that there is significant difference in almost all gesture pairs analysed. Each cell indicates the comparison of two gestures. There is no significant difference in pairs with a *p*-value greater than 0.001, as such there is no difference in the degree of difficultly between the gestures paired in the list below:

- Tap/Touch and hold/Slide
- Touch and hold/Slide.
- Touch and slide/Pinch/Stretch.
- Double tap/Pinch
- Rotate with fingers of both hands/Join with fingers of both hands.
- Close fist/Spread fingers/Stop with hand
- Spread fingers/Rotate with the hand
- Move with the hand/Rotate with the hand

In contrast, the use of both hands presents a different degree of difficulty, whatever the gesture. Table A1, which can be found in the Appendix A of this paper, contains pair-wise gesture comparisons to test whether the success rate was independent of gesture (complete comparison). This table is of interest to readers wishing to know the significance between two gestures of different groups.

This section may be divided by subheadings. It should provide a concise and precise description of the experimental results, their interpretation as well as the experimental conclusions that can be drawn.

### 5.2. Success Rate by Gender, Socioeconomic Status, and Type of Down Syndrome

**Figure 4:**
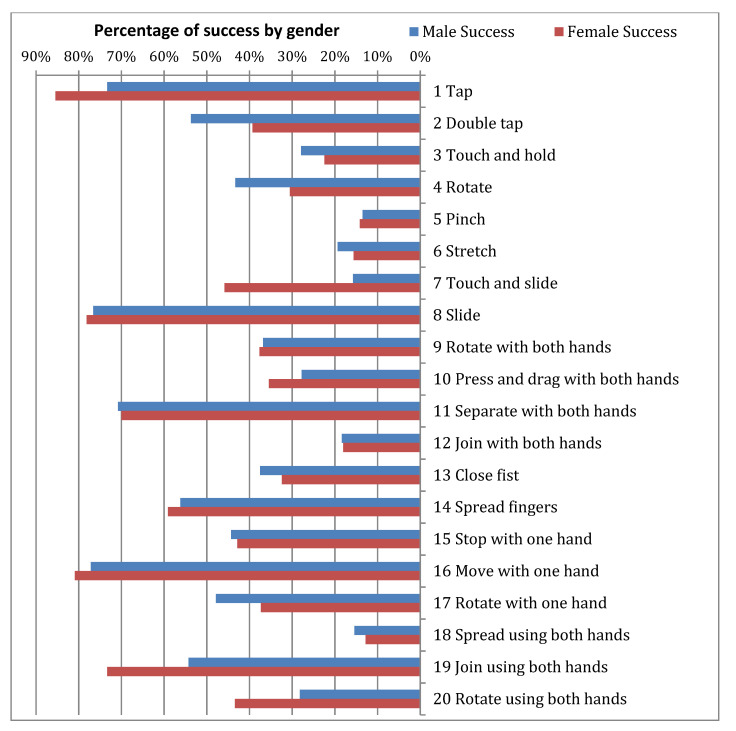
Success rate and failure rate by gender.

**Figure 5:**
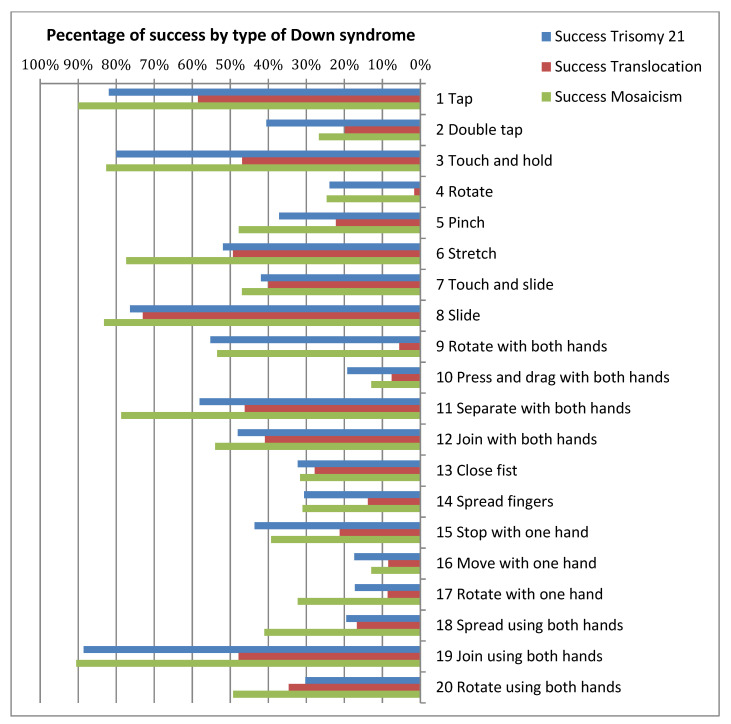
Success rate and failure rate by type of Down syndrome.

**Figure 6:**
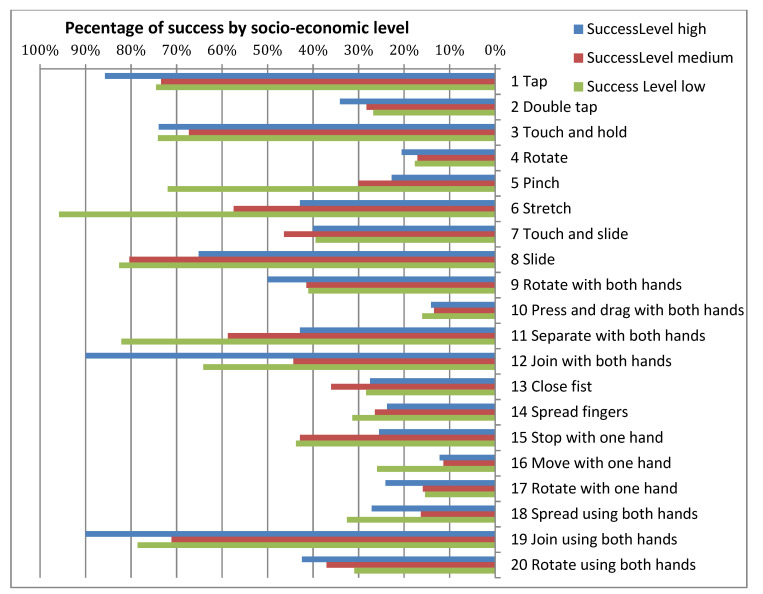
Success rate and failure rate by socioeconomic status.

Figure 4, Figure 5 and Figure 6 show the success/failure rate of each gesture by gender, socioeconomic status and type of Down syndrome, respectively.

The objective is to establish whether the success rate of a gesture is dictated by any of the following factors: *gender*, *socioeconomic status*, or *type of Down syndrome*. Pearson’s chi-square tests were conducted on each gesture to determine the independence of success from these three qualitative factors.

In the column for *Gender* in Table 10, it is possible to observe that the majority of gestures do not affect the success rate; in other words, male of female participants with DS do not find it difficult to execute these gestures. From the results displayed in this column, it can be observed that four gestures are significantly influenced by the factor *Gender* (gestures 11, 12, 13, and 18). From a statistical standpoint, this would be sufficient to argue that the success rate of a gesture is indeed influenced by gender, however, as data indicates that gender only affects the success rate of four out of a total of 20 gestures it would also be possible to accept the null hypothesis (Ho_3_) of research hypothesis HR3. However, strictly speaking, the null hypothesis is rejected given that there is significant difference for gender in four gestures. For *Socioeconomic Status* the results were uniform. For the same reason mentioned above, it could be argued that overall there is no significant difference, and as such the null hypothesis (Ho_6_) of research hypothesis HR6 could be accepted. It could be assumed that users possessing touchscreen devices i.e., smartphones, which are items that the middle and upper classes are more able to afford, would demonstrate greater dexterity due to increased practice. However, analysis demonstrated that although only five gestures showed significant difference for *Socioeconomic Status* (gestures 5, 6, 8, 11, and 16) the null hypothesis (Ho_6_) for research hypothesis HR6 must be rejected given that significant difference does in fact exist for *Socioeconomic Status* in five gestures. In the column displaying results for *type of Down syndrome* it can be observed that this is clearly a significant factor as almost all the *p*-values are under 0.05, with the exception of six gestures that do not show significant difference, theses being: 7, 8, 12, 13, 15, and 16. As such, the null hypothesis (Ho_5_) of research hypothesis HR5 is accepted. 

For this reason, the analysis of the three variables demonstrated that the *type of Down syndrome* does influence the success rate of gestures, and to a lesser extent so do *gender* and *socioeconomic status*. Table 10 is completed with an analysis of the interactions between the variables *Gender-Socioeconomic Status*, *Gender-Type of Down Syndrome*, and *Socioeconomic Status-Type of Down Syndrome*. With the exceptions of gestures 5 and 7, results show that there is significant difference in *gender* and *socioeconomic status*, meaning that there is significant difference in the function of socioeconomic status and whether someone is male or female; in the contingency tables obtained from calculations it is possible to observe that females of a lower socioeconomic status commit a greater number of errors. In the interaction between *gender* and type of Down syndrome 50% of the gestures analysed (10 gestures) reveal ‘significant difference’. When observing the data, it can be seen that more errors are committed by female participants regardless the type of Down syndrome they live with. It can therefore be concluded that women with Down syndrome encounter greater difficulties when performing touch gestures. The interaction between *socioeconomic status* and *type of Down syndrome* indicates there is significant different; at all social levels it is clear the variable *type of Down syndrome* influences the success rate of gesture execution as *p*-value = 0.000.

In conclusion, this analysis of success rates based on the study variables has demonstrated that although the factor *gender* does not affect all gestures, research hypothesis HR3 is rejected because gender does in fact affect some of the gestures. The null hypothesis (Ho_6_) of research hypothesis HR6 is rejected and the null hypothesis (Ho_5_) of research hypothesis HR5 is accepted for the same reasons, as both *socioeconomic Status* and *type of Down Syndrome* affect the success rate of a gesture. Whilst this analysis is valuable, the detailed data provided in the tables specifies the effect, or lack thereof, of each of these variables on each gesture.

### 5.3. Completion Time—ANOVA

An initial calculation related to the type of gesture and completion time, using ANOVA and the dependent variable *Timer* and independent variable *Type of gesture* (F = 51.010, *p*-value = 0.000) indicates that there is significant difference in the duration of different types of gestures. The null hypothesis (Ho_2_) of research hypothesis HR2 is accepted: *Gesture difficulty is correlated with completion time.*

**Figure 7:**
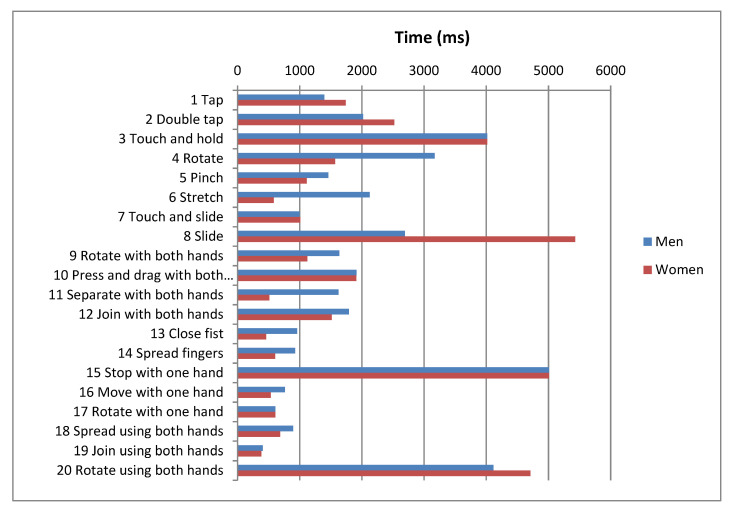
Mean completion times for each gesture by gender.

**Figure 8:**
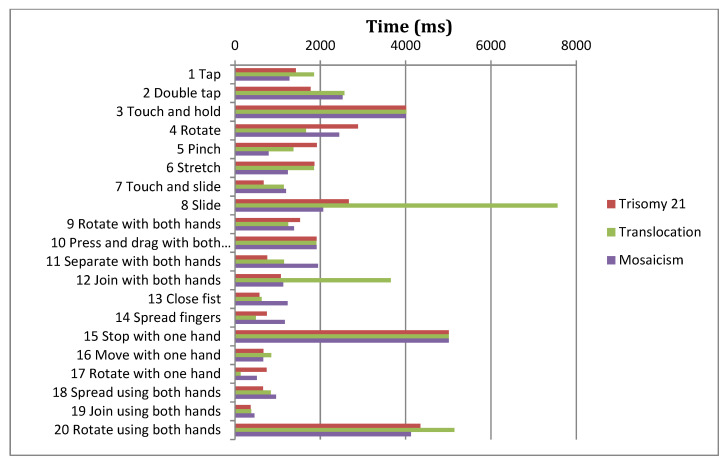
Mean completion times for each gesture by type of Down syndrome.

**Figure 9:**
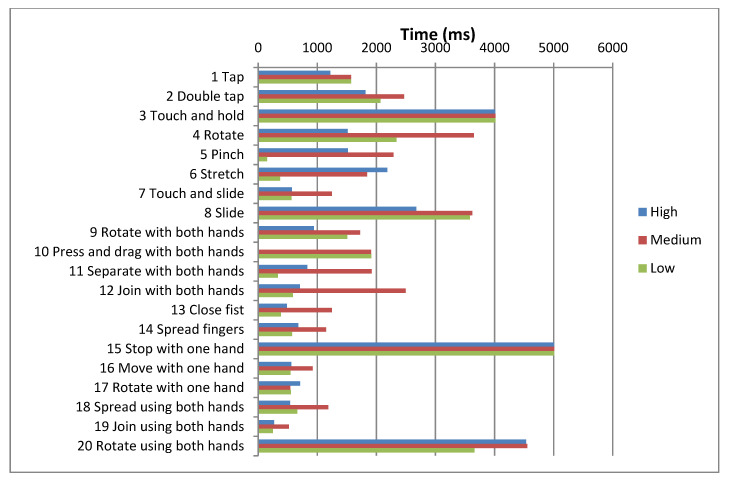
Mean completion times for each gesture by socioeconomic status.

The research project and corresponding study were performed in order to establish the influence of the variables *gender*, *type of Down syndrome* and *socioeconomic status* on the completion times of gestures. For this analysis the authors considered only including gestures that were successfully completed. Figure 7, Figure 8 and Figure 9 display the average times for each of the gestures and for each of the variables being researched.

Two-way ANOVA is applied with the independent variable of *gender*, *type of Down syndrome* and *socioeconomic status*, and the dependent variable of *completion time* (Table 11). This shows that for three gestures (11, 15, 19) completion time is influenced by gender, whereas in the majority of gestures completion time is not significantly influenced by gender (*p*-value > 0.05). As such, research hypothesis HR4 is rejected and therefore the null hypothesis Ho_4_ is also rejected; completion time is gender dependent. It has also been demonstrated that completion time is significantly influenced (*p*-value < 0.05) by the type of Down syndrome (gestures: 2, 3, 8, 10, 13, 14, 15, 16, 17, 18, and 19) and by socioeconomic status (gestures: 2, 4, 5, 6, 7, 8, 11, 12, 13, 14, 15, 16, 18, and 19), as such research hypotheses HR7 and HR8 are accepted. 

Analysis also reveals that completion time is significantly influenced by the interaction of the following factors: *Gender-Type of Down syndrome*, *Gender-Socioeconomic Status*, and *Type of Down Syndrome-Socioeconomic Status*.

The observation of data in this analysis indicates that male participants perform gestures faster than female participants, and that individuals of a higher socioeconomic status perform gestures faster than those of a lower socioeconomic status. In general, there are no differences in the completion times between *Type of Down Syndrome-Gender*, although there are for *Type of Down Syndrome-Socioeconomic Status*. As such, it appears that the variables *socioeconomic status* and *gender* do in fact affect gesture completion, and thus success rates.

### 5.4. Observational Findings/Qualitative Results

This section contains valuable information regarding participant behaviour during the study. In addition to providing instructions and assistance to participants during tasks the moderator took notes of any difficulties that influenced whether a task was successful or unsuccessful, as well as any issues that caused participants to take longer to complete a given task.

A common habit shared by all participants was that of pressing the touchscreen forcefully with one finger and holding the finger down in the same position over an extended period of time. This did not give rise to too many complications with gestures such as the ‘Tap’ gesture, although it did pose more of a problem when participants came to use the ‘Double Tap’ gesture and translated into a greater number of ‘fails’. The action of pressing down with force over a continued period of time meant that the device interpreted the action as a ‘Drag’ gesture instead of a ‘Tap’ gesture.

It was observed that certain gestures were often not fully completed: in the case of one-handed finger gestures requiring participants to drag an object over a specific distance, i.e., *Touch and hold,* participants would stop dragging too soon; and in the case of gestures involving sliding or rotation motions, i.e., *Pinch, Rotate, Stretch, Slide, Touch and slide,* participants would abandon a gesture before completing a task when force was required, rather than just positioning fingers on the screen and sliding them. This led to much frustration among participants, as no matter how hard they tried they were unable to finish a task as a consequence of ending a gesture before the task was complete, and thus a ‘failed’ gesture being recorded.

In the case of gestures that require the use of two fingers, success rates were higher. However, it was observed that there was a lack of coordination when it came to getting both fingers to the same destination, e.g., in the case of *Join*, or when separating both fingers. This lack of coordination was most evident in the *Rotate* gesture using two fingers. For the gesture *Press and drag*, the challenge proved to be understanding the instructions: participants would instinctively make the *Separate* gesture, and they felt the need to concentrate hard in order to be mentally alert enough to hold one finger in place whilst moving the other. 

All of the physical or observable incidences that were detected are the consequence of the physical traits found in individuals with DS, such as rigidity in the extremities, difficulty moving arms, or difficulty with concentrating.

Regarding interactions involving the *Double tap* gesture, it was observed that cognitive complexity of decision-making is involved. Participants were seen repeatedly tapping the screen, up to four or five times, even once an object had disappeared.

Regarding gestures performed with the entire hand, it was observed that some gestures were easier to perform and the success rate for these was almost perfect, i.e., *Stop*, *Move*, and *Rotate with hand*. The gestures *Close fist* and *Spread fingers* proved more difficult. These consisted of joining or separating all five fingers of one hand on the touchscreen. As the fingers of individuals with DS are shorter, more curved, and more rigid, the act of opening and closing the fingers of the hand on a screen’s surface proved to be no easy feat for them.

Regarding gestures with two hands, *Spread* and *Rotate* had a high failure rate as a consequence of a lack of coordination between the two hands when moving at the same time. However the gesture *Join* using both hands had a higher success rate.

It was also observed that female participants showed greater patience when performing tasks and older individuals made efforts to improve on subsequent attempts. The youngest individuals found it hardest to focus their attention and concentrate on the task at hand, and, even though the tasks involved “playing games” they were easily distracted. 

## 6. Discussion

### 6.1. UX Considerations

Based on the results obtained, individuals with Down syndrome are able to perform all of the proposed touch gestures (one-handed finger gestures, two-handed finger gestures, one hand touch gestures, and two hand touch gestures) although the success rates and completion times for each of these varies considerably.

This study analyses the differences between gestures in terms of execution success rates and completion times. For execution success rates, higher success rates were obtained for the gestures *Slide, Tap, Touch and slide, Stretch, Separate,* and *Join*. When performing pairwise comparisons of gestures, it was found that certain pairs had the same success rates: Tap/Touch and Hold; Tap/Slide; Double tap/Pinch; Touch and hold/Stretch; Rotation 2 hands/Join 2 hands; Touch and hold/Slide; Pinch/Touch and Slide.

Comparative analogous research was performed to study differences in completion times. It was found that no differences exist between certain gesture pairs (Stretch/Pinch; Stretch/Touch and slide; Separate 2 hands/Join 2 hands; Move with the hand/Rotate with the hand). The comparisons presented in this paper will help developers select suitable gestures when designing an application.

What is more, the data presented in this paper reveals issues that require further discussion. In all instances, verbal instructions proved insufficient and the moderator needed to physically demonstrate how to perform the gestures to participants so they could understand what was required of them. For their part, participants were more interested in completing the tasks themselves than in performing the gesture, as such researchers observed that they would find and use shortcuts to complete the tasks. For example, some individuals would use the fingers on both hands, despite having received instructions to only use two fingers from the same hand.

During the *Tap* gesture, some participants would place both hands on the edge of the tablet and swap indiscriminately between the thumbs on either their left or right hand when performing an action in order to use the thumb closest to the object on screen. The *Double tap* gesture tended to lead to errors. It was observed that the majority of participants waited for too long between the first and second tap, thus producing a failed or ‘false’ gesture. This led to a sense of frustration among users, even causing one participant to forcefully press down on the object. Other individuals reacted by tapping the screen 3 or 4 times instead of twice, despite the fact that the object had already disappeared. Thus, developers are advised to use the *Tap* gesture rather than the *Double tap* gesture. This issue could be resolved by adapting the programming behind the gesture to the motor skills of these individuals, eliminating lag between taps or, equally, by ignoring subsequent taps once a task is completed.

In the task created to elicit the *Hold* gesture participants had to stop the Sun as it crossed the sky. Two problems were observed: firstly, instead of stopping the Sun itself some users would use the gesture slightly behind the object as it travelled its path across the screen; secondly, some users stopped the object itself, but did not continue the gesture for long enough. An audio cue was provided to indicate when the task had been successfully completed, however some participants would stop the *Hold* gesture before hearing this audio cue. It is recommended that developers avoid using moving objects if they intend to use this gesture.

The *Rotate* gesture is not performed in one single smooth movement, but instead in several steps. One participant used two fingers, but did not separate them enough and the gesture was detected as a single finger, thus a failed or ‘false’ gesture was recorded. On occasion, other participants lifted their hand off the tablet and thus a failed or ‘false’ gesture was recorded. They quickly learnt that they had to keep a least one finger on the screen, and did so. It was also observed that participants always performed the *Rotate* gesture in a clockwise fashion, most likely due to being right-handed, and did so even when a counter clockwise rotation would have been shorter. Developers should include visual cues indicating the recommended direction for performing the *Rotate* gesture.

For the *Zoom in* and *Zoom out* gestures, participants were only able to perform 75% of either gesture, never the complete gesture, due to having short fingers. Some participants repeatedly used fingers from both hands, ignoring repeated instructions from the moderator informing them that the gesture had to be performed using the fingers from one hand only.

For the *Slide* gesture the majority of participants performed it without difficulty. However, in the case of two individuals it was observed that they did not start the gesture on the ball itself, but instead they started the gesture just in front of the object causing a failed gesture to be recorded. Nonetheless, it is believed that this problem comes down to how the task was designed. Developers should keep context in mind to ensure that the gesture is started on the object that has to be moved.

The gesture *Press and drag* proved to be the most complicated for participants to perform. Some individuals tried to perform symmetrical gestures using both hands. In other words, if the finger of the left hand performed the *Press*, they mirrored this with the finger of the right hand and, consequently, they stopped pressing on the screen when they then had to perform the *Drag* action. The easiest way to perform this gesture is to press with the left finger and drag with the right finger, although some individuals did this back to front which caused them to clumsily cross one hand over the other when trying to drag the object.

Most participants could perform the *Separate* gesture without problem. However, they did not execute the action in one smooth go. Instead they would perform the first half of the gesture and then the second.

The *Close fist* gesture did not present participants with any difficulties.

Participants could perform the *Spread fingers* gesture, although their fingers slipped and it generally took them a long time to perform.

For the one-handed *Stop* gesture, there was one participant who would stop the object with the entire hand but then lift all but two fingers of the screen. Nonetheless, we recommending using the one-handed gesture as it proved easier for participants.

For the *Move with hand* gesture it was observed that despite users performing the gesture correctly, the tablet did not detect all fingers. It was discovered that performing the gesture with just three fingers was better than performing it with all five fingers. 

For the gesture *Spread with both hands*, participants found it very complicated to use when interacting with small objects.

The *Join using both hands* gesture did not present participants with any difficulties.

For the *Rotate using both hands* gesture many participants preferred using only one hand. It became evident that if an object was very small users would place both hands within the object and it became difficult to rotate, but with larger objects the task proved easier.

The results and observations obtained in this study enable designers to follow a Design for All approach that supports the special needs of people with DS. The interface can be adjusted to the needs and development level of these users in an adaptive way.

### 6.2. Gender, Social Status and Type of Down Syndrome

Several pieces of research analyse the effects of gender given that this variable can influence a study. In usability studies in particular, gender is a variable that must be taken into account given that behaviours and use preferences may differ between males and females (e.g., colours, typography, saturation of information, type of image) [77]. In this particular study that has been performed specifically with individuals with Down syndrome no significant difference was identified for success rates or completion times by *gender* when performing touch gestures on a touchscreen, thus it is possible to argue that the difficulty experienced when performing a gesture (dependant on a unique set of intellectual and physical limitations) and the success rates for gestures are the same for both men and women. The authors of this paper had believed that the socioeconomic status of participants would prove to be a key factor in the success rate of gestures, given that individuals from High or Medium socioeconomic levels have more access to touchscreen devices. However this study has demonstrated that it is not a significant factor, as neither success rates nor completion times show significant difference by *socioeconomic status*. From a biological standpoint, there are three types of Down syndrome. Following analysis, significant difference was identified for success rates and completion times by *type of Down syndrome*. Individuals with Mosaicism achieved higher success rates and faster completion times than individuals with Trisomy 21 and translocation DS. No differences were identified between individuals with Trisomy 21 and translocation DS.

Far from attempting to establish intellectual differences between different types of Down syndrome, this paper is merely highlighting the findings obtained from this particular study for the purpose of designing apps that use touch gestures that everyone finds easy and intuitive to use.

Numerous studies indicate that differences in intelligence quotient (IQ) between individuals with Down syndrome are similar to those found in any other group of individuals without learning difficulties [78]. As with any other human characteristic, IQ levels result from genetic inheritance and chance. Differences that exist between individuals are exacerbated to a greater or lesser extent by an individual’s own unique set of personal characteristics, their life experiences and, above all, external stimulation [79]. Every single individual is unique. Everyone has their own personal strengths and weaknesses, and each of us will live different life experiences. The same is true of individuals with Down syndrome. It has been demonstrated that the early stimulation newborns are exposed to in the first few days of life has a significant positive impact on the lives of individuals with learning difficulties [80]. Furthermore, as would be true for anyone, those living with Down syndrome are now achieving things that until recently were considered unimaginable as a result of changing environmental and social expectations, being given opportunities to participate in common areas, and receiving support from others.

### 6.3. Designing Multi-Touch Application for SD (or for all)

This work highlights the capacity of individuals with Down syndrome, or likewise any individual with a certain degree of physical or intellectual disability, to perform touch gestures on a touchscreen. The observations and findings from this study indicate which gestures are deemed most suitable when trying to ensure high success rates and rapid gesture execution.

To support this work a visual guidebook has been created (see Appendix B) to assist application developers and manufacturers of touchscreen devices. The guidebook lists available gestures together with their characteristics, and includes recommendations on which gestures should be considered as part of a Design for All approach to ensure app design and development is focused on as many users as possible.

## 7. Conclusions

This study has evaluated a total of 20 touchscreen gestures performed using one-handed finger gestures, two-handed touch gestures, one hand touch gestures, and two hand touch gestures with individuals living with Down syndrome to determine if differences are present in terms of ease of use and execution times of said gestures.

The study is founded on the premise of Design for All. From this standpoint it has been argued that design processes should take into consideration the needs of users with Down syndrome to avoid frustrations as a result of poor design, or at least understand what the most suitable design choices and operations are for this group of individuals.

This study’s results reveal that individuals with Down syndrome can perform all the multi-touch gestures evaluated, although success rates vary considerably depending on the gesture in question. The evaluated gestures can be used in future designs of touchscreen apps targeting this group of individuals even though there are differences in execution times and success rates between some participants when compared against each other. Thus, this work can assist developers in discerning between gestures and help them to pick the most suitable gestures based on success rate or execution time, which in turn will help them to create apps with more natural and intuitive interactions.

The study does not find statistically significant results for success rates by the variables of *gender* or, democratically, *socioeconomic status* (only four out of 20 gestures showed significant differences). However, the variable *type of Down syndrome* did affect success rates. Table 12 shows the acceptance/rejection conclusion of the hypotheses studied in this study.

As a future line of research the authors wish to explore the influence of success rate and execution time following the principles of interaction of Fitts’s Law.

## Figures and Tables

**Table 1:** Distribution of participants by gender, socioeconomic status and type of Down syndrome (DS): Trisomy 21-T21; Translocation-TR; Mosaicism-MOS.

		Socioeconomic Status			Type of DS		
	No.	High	Med.	Low	T21	TR	MOS
Male	13	3	7	3	6	4	3
Female	5	2	2	1	2	2	1
Total	18	5	9	4	8	6	4

**Table 2:** One-handed finger gestures: tasks used to elicit and measure gestures.

Goal	Gesture	Figure	Task	Task Instructions
Select object	1. Tap	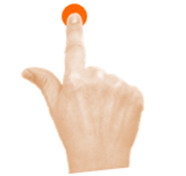	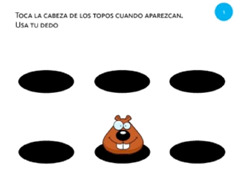	Using one finger, tap the mole with your finger.
Select object	2. Double tap	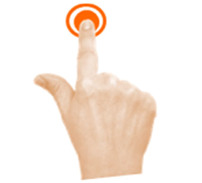	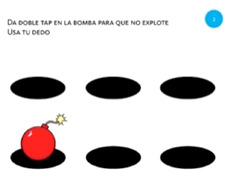	Using one finger, double tap the bomb to stop it exploding.
Select object	3. Touch and hold	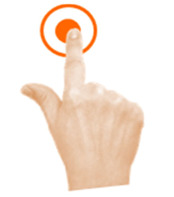	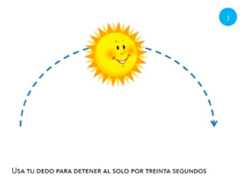	Using one finger, stop the sun until you hear the beep.
Rotate object	4. Rotate	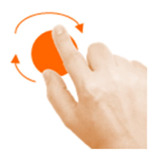	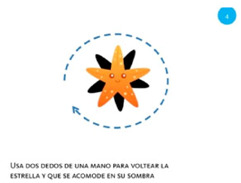	Using two fingers on the same hand, rotate the star until it covers its shadow.
Scale object	5. Pinch	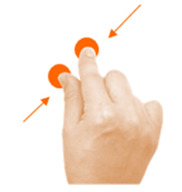	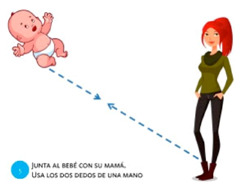	Using two fingers from the same hand, reunite the baby with its mother.
Scale object	6. Stretch	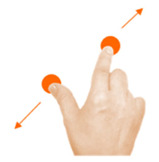	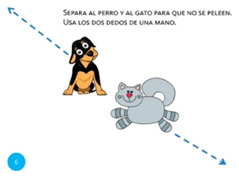	Using two fingers from the same hand, stop the cat and dog from fighting.
Move object	7. Touch and slide	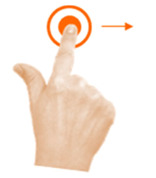	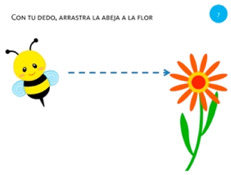	Using one finger, drag the bee to the flower.
Move object	8. Slide	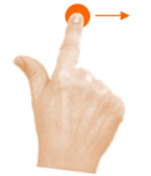	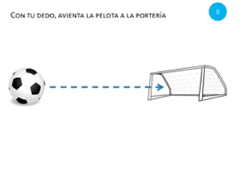	Using one finger, move the football into the goal.

**Table 3:** Two-handed touch gestures using fingers: tasks used to elicit and measure gestures.

Goal	Gesture	Figure	Task	Task Instructions
Rotate object	9. Rotate	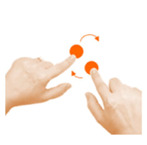	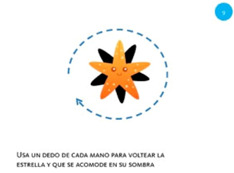	Using one finger from each hand, rotate the star until it covers its shadow.
Move object	10. Press and drag	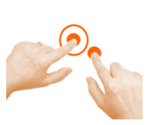	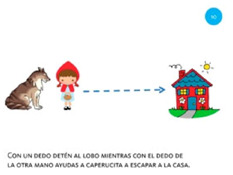	Using one finger from your left hand, stop the wolf. Using one finger from your right hand, help Little Red Riding Hood get home safely.
Move object	11. Separate	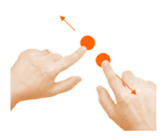	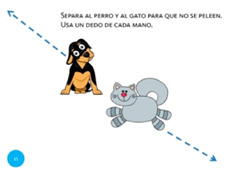	Using one finger from each hand, stop the cat and dog from fighting.
Move object	12. Join	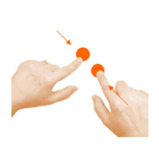	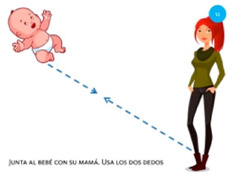	Using one finger from each hand, reunite the baby with its mother.

**Table 4:** One-handed touch gestures using the whole hand: tasks used to elicit and measure gestures.

Goal	Gesture	Figure	Tasks	Task Instructions
Scale object	13. Close fist	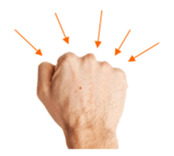	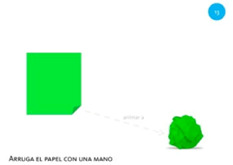	Using one hand, make a paper ball from the sheet of paper.
Scale object	14. Spread fingers	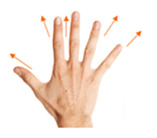	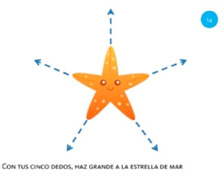	Using all five fingers from one hand, make the starfish bigger.
Select object	15. Stop with hand	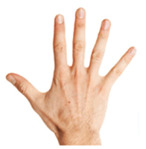	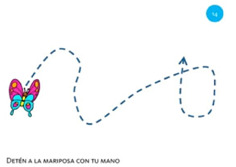	Using one hand, stop the butterfly.
Move object	16. Move with hand	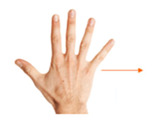	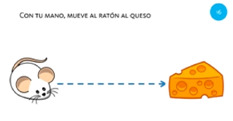	Using one hand, drag the mouse to the cheese.
Rotate object	17. Rotate	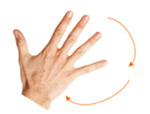	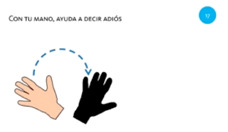	Using one hand, wave goodbye.

**Table 5:** Two-handed touch gestures using whole hand: tasks used to elicit and measure gestures.

Goal	Gesture	Figure	Tasks	Task Instructions
Move object	18. Spread using both hands	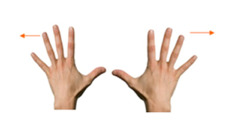	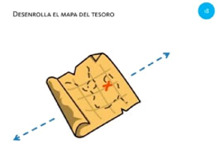	Using both hands, unroll the treasure map.
Move object	19. Join using both hands	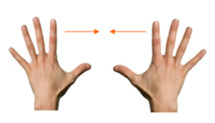	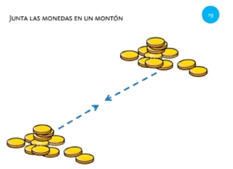	Using both hands, join the two piles of coins.
Rotate object	20. Rotate using both hands	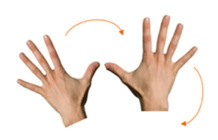	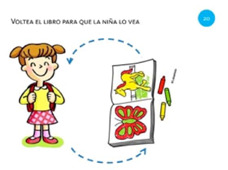	Using both hands, rotate the book so the girl can read it.

**Table 6:** Comparison of gestures (fingers—one hand) by success with Pearson’s chi-squared test of independence χ² (DoF = 1, N = 18). Bonferroni *p*-value *p* < 0.05/28 = 0.00178 *, *p* < 0.001 **.

Success// Completion Time	1	2	3	4	5	6	7	8
**1. Tap**	-	0.000 **	0.273	0.000 **	0.000 **	0.000 **	0.000 **	0.573
**2. Double tap**	0.000	-	0.000 **	0.001 *	0.060	0.000 **	0.000 **	0.000 **
**3. Touch and hold**	0.000	0.000	-	0.000 **	0.000 **	0.019	0.000 **	0.085
**4. Rotate**	0.000	0.000	0.000	-	0.000 *	0.000 **	0.000 **	0.000 **
**5. Pinch**	0.004	0.000	0.000	0.000	-	0.000 **	0.050	0.000 **
**6. Stretch**	0.001	0.000	0.000	0.000	0.405	-	0.002	0.000 **
**7. Touch and slide**	0.000	0.000	0.000	0.000	0.004	0.056	-	0.000 **
**8. Slide**	0.000	0.000	0.000	0.000	0.000	0.000	0.000	-

(* *p*-value < 0.00178; ** *p*-value < 0.001).

**Table 7:** Comparison of gestures (fingers—two hands) by success with Pearson’s chi-squared test of independence χ² (DoF = 1, N = 18). Bonferroni *p*-value *p* < 0.05/6 = 0.008 *, *p* < 0.001 **.

Success// Completion Time	9	10	11	12
**9. Rotate—2 hands**	-	0.000 **	0.001 *	0.420
**10. Press and drag—2 hands**	0.000	-	0.000 **	0.000 **
**11. Separate—2 hands**	0.000	0.000	-	0.005 *
**12. Join—2 hands**	0.000	0.000	0.313	-

(* *p*-value < 0.008; ** *p*-value < 0.001).

**Table 8:** Comparison of gestures (one hand—whole) by success with Pearson’s chi-squared test of independence χ² (DoF = 1, N = 18). Bonferroni *p*-value *p* < 0.05/10 = 0.005 *, *p* < 0.001 **.

Success// Completion Time	13	14	15	16	17
**13. Close fist**	-	0.258	0.164	0.000 **	0.002 *
**14. Spread fingers**	0.546	-	0.019	0.000 **	0.038
**15. Stop with hand**	0.000		-	0.000 **	0.000 **
**16. Move with hand**	0.000	0.000	0.000	-	0.168
**17. Rotate with hand**	0.000	0.000	0.000	0.523	-

(* *p*-value < 0.005; ** *p*-value < 0.001).

**Table 9:** Comparison of gestures (two hands—whole) by success with Pearson’s chi-squared test of independence χ² (DoF = 1, N = 18). Bonferroni *p*-value *p* < 0.05/3 = 0.017 *, *p* < 0.001 **.

Success// Completion Time	18	19	20
**18. Spread using both hands**	-	0.000 **	0.003 *
**19. Join using both hands**	0.000	-	0.000 **
**20. Rotate using both hands**	0.000	0.004	-

(* *p*-value < 0.017; ** *p*-value < 0.001).

**Table 10:** Statistic of Pearson’s chi-squared test. F-statistic from the success analysis.

ID Gest	Gender			Socioeconomic Status			Type of Down Syndrome			Gen-ES		Gen-TD		ES-TD	
	DoF	χ²	*p*-Val	DoF	χ²	*p*-Val	DoF	χ²	*p*-Val	χ²	*p*-Val	χ²	*p*-Val	χ²	*p*-Val
**1**	1	2.933	0.087	2	2.652	0.266	2	18.062	**0.000**	29.515	**0.000**	3.459	0.177	93.554	**0.000**
**2**	1	2.317	0.128	2	1.798	0.407	2	14.719	**0.001**	57.711	**0.000**	10.163	**0.006**	192.63	**0.000**
**3**	1	0.007	0.933	2	0.570	0.752	2	12.373	**0.002**	18.452	**0.000**	0.618	0.734	42.078	**0.000**
**4**	1	0.003	0.958	2	0.399	0.819	2	15.897	**0.000**	17.329	**0.000**	38.558	**0.000**	84.117	**0.000**
**5**	1	0.613	0.434	2	39.640	**0.000**	2	12.108	**0.002**	3.443	0.179	5.970	0.051	115.628	**0.000**
**6**	1	0.163	0.686	2	20.493	**0.000**	2	11.774	**0.003**	8.517	**0.014**	0.659	0.719	99.937	**0.000**
**7**	1	0.073	0.787	2	1.597	0.450	2	1.246	0.536	5.166	0.076	36.767	**0.000**	94.177	**0.000**
**8**	1	0.913	0.339	2	10.478	**0.005**	2	5.412	0.067	20.469	**0.000**	4.842	0.089	191.607	**0.000**
**9**	1	1.775	0.183	2	0.923	0.630	2	27.175	**0.000**	12.767	**0.002**	0.696	0.706	60.416	**0.000**
**10**	1	0.853	0.356	2	0.557	0.757	2	11.424	**0.003**	14.408	**0.000**	10.157	**0.006**	246.112	**0.000**
**11**	1	7.937	**0.005**	2	18.672	**0.000**	2	14.263	**0.001**	19.255	**0.000**	0.296	0.862	127.597	**0.000**
**12**	1	5.321	**0.021**	2	5.081	0.079	2	2.332	0.312	33.630	**0.000**	5.039	0.081	93.451	**0.000**
**13**	1	4.611	**0.032**	2	2.109	0.348	2	0.363	0.834	8.962	**0.011**	18.336	**0.000**	108.402	**0.000**
**14**	1	0.684	0.408	2	0.930	0.628	2	6.440	**0.040**	39.687	**0.000**	11.292	**0.004**	105.358	**0.000**
**15**	1	2.782	0.095	2	4.701	0.095	2	5.024	0.081	4.611	**0.000**	9.294	**0.010**	66.710	**0.000**
**16**	1	0.031	0.860	2	7.951	**0.019**	2	4.211	0.122	22.109	**0.000**	26.185	**0.000**	162.229	**0.000**
**17**	1	0.396	0.529	2	1.839	0.399	2	6.404	**0.041**	42.900	**0.000**	1.160	0.560	58.761	**0.000**
**18**	1	18.346	**0.000**	2	5.078	0.079	2	8.845	**0.012**	42.405	**0.000**	15.198	**0.001**	62.605	**0.000**
**19**	1	0.025	0.874	2	2.826	0.243	2	15.956	**0.000**	18.357	**0.000**	0.149	0.923	24.626	**0.000**
**20**	1	0.015	0.902	2	1.705	0.426	2	6.483	**0.039**	46.578	**0.000**	18.119	**0.000**	55.261	**0.000**

In Bold, *p*-values < 0.05. This means that the gesture has significant difference for the column variable.

**Table 11:** Statistic of Pearson’s chi-squared test. F-statistic from the completion time analysis.

ID Gest		Gender			Socioeconomic Status		Type of Down Syndrome			Gen-ES		Gen-TD		ES-TD	
	DoF	F	*p*-Val	DoF	F	*p*-Val	F	*p*-Val	DoF	F	*p*-Val	F	*p*-Val	F	*p*-Val
**1**	1	0.042	0.838	2	2.525	0.083	2.899	0.058	4	0.427	0.514	0.787	0.375	8.620	**0.004**
**2**	1	0.001	0.982	2	8,379	**0.000**	9.238	**0.000**	4	0.687	0.408	0.486	0.610	70.597	**0.000**
**3**	1	1.389	0.241	2	0.605	0.548	16.288	**0.000**	4	4.743	**0.032**	1.341	0,248	0.442	0.508
**4**	1	0.030	0.863	2	0.127	**0.000**	0.924	0.398	4	0.034	0.854	0.160	0.689	9.081	**0.003**
**5**	1	1.137	0.287	2	14.003	**0.000**	2.190	0.114	4	0.162	0.688	12.784	**0.000**	0.074	0.785
**6**	1	2.586	0.109	2	3.774	**0.024**	1.009	0.366	4	2.014	0.157	5.250	**0.023**	0.354	0.552
**7**	1	3.244	0.073	2	3.089	**0.047**	0.415	0.661	4	0.090	0.765	0.048	0.826	6.932	**0.009**
**8**	1	12.074	**0.001**	2	3.654	**0.027**	52.245	**0.000**	4	12.047	**0.001**	31.646	**0.000**	2.137	0.144
**9**	1	0.123	0.726	2	0.491	0.613	1.702	0.186	4	0.190	0.827	0.984	0.376	0.277	0.758
**10**	1	0.023	0.879	2	0.016	0.985	5.184	**0.006**	4	3.293	0.070	1.688	0.194	3.259	0.072
**11**	1	14.607	**0.000**	2	9.952	**0.000**	1.947	0.145	4	0.788	0.376	5.217	**0.023**	22.004	**0.000**
**12**	1	1.185	0.277	2	4.954	**0.008**	1.360	0.259	4	0.300	0.584	2.809	0.095	8.814	**0.003**
**13**	1	0.120	0.729	2	8.540	**0.000**	8,365	**0.000**	4	0.716	0.398	0.449	0.503	6.109	**0.003**
**14**	1	19.335	**0.000**	2	17.607	**0.000**	16.403	**0.000**	4	0.387	0.534	2.994	0.085	8.435	**0.000**
**15**	1	0.013	0.911	2	9.213	**0.000**	11.690	**0.000**	4	4.638	**0.033**	0.047	0.834	1.024	0.313
**16**	1	1.009	0.316	2	16.019	**0.000**	14.374	**0.000**	4	1.346	0.247	0.766	0.382	6.255	**0.002**
**17**	1	1.052	0.306	2	0.099	0.905	3.898	**0.022**	4	18.170	**0.000**	0.469	0.494	0.674	0.511
**18**	1	3.588	0.060	2	5.835	**0.003**	15.691	**0.000**	4	0.049	0.826	0.615	0.559	5.110	**0.025**
**19**	1	10.940	**0.001**	2	13.300	**0.000**	10.328	**0.000**	4	0.012	0.901	1.945	0.143	0.284	0.743
**20**	1	0.042	0.837	2	1.079	0.342	0.204	0.815	4	0.780	0.378	1.191	0.288	7.674	**0.006**

In Bold, *p*-values < 0.05. This means that the gesture has significant difference for the column variable.

**Table 12:** Summary of accepted/rejected hypotheses.

Hypothesis	Conclusions
HR1. Touchscreen gestures pose different levels of difficulty for individuals living with Down syndrome. Ho_1_: The gestures analysed have the same degree of difficulty.	Ho_1_ is rejected. The statement of HR1 is accepted.
HR2. The gesture used has an effect on completion time. Ho_2_: Gesture difficulty is related to completion time.	Ho_2_ is accepted. The statement of HR2 is accepted.
HR3. Gender has an effect on the success rate of a gesture. Ho_3_: The gender of a person with DS does not influence the success rate of a gesture.	Ho_3_ is accepted. The statement of HR3 is rejected.
HR4. Gender has an effect on the completion time of a gesture. Ho_4_: The gender of a person with DS does not influence the completion time of a gesture.	Ho_4_ is rejected. The statement of HR4 is rejected.
HR5. The type of Down syndrome has an effect on the success rate of a gesture. Ho_5_: The type of Down syndrome influences the success rate of a gesture.	Ho_5_ is accepted. The statement of HR5 is accepted.
HR6. Socioeconomic status has an effect on the success rate of a gesture. Ho_6_: Socioeconomic status does not influence the success rate of a gesture.	Ho_6_ is accepted. The statement of HR6 is rejected.
HR7. The type of Down syndrome has an effect on the completion time of a gesture. Ho_7_: The completion time of a gesture is not influenced by the type of Down syndrome.	Ho_7_ is rejected. The statement of HR7 is accepted.
HR8. Socioeconomic status has an effect on the completion time of a gesture. Ho_8_: Socioeconomic status influences gesture completion time.	Ho_8_ is accepted. The statement of HR8 is accepted.

## Data Availability

The data presented in this study are available in the paper.

## Appendix A

**Table A1:** Comparison of touch gestures by success with Pearson’s chi-squared test of independence χ² (DoF = 1, N = 18). *p*-values.

Success Completion Time	1	2	3	4	5	6	7	8	9	10	11	12	13	14	15	16	17	18	19	20
**1. Tap**	-	0.000	0.273	0.000	0.000	0.000	0.000	0.573	0.000	0.000	0.000	0.000	0.000	0.000	0.000	0.000	0.000	0.000	0.866	0.000
**2. Double tap**	0.000	-	0.000	0.001	0.060	0.000	0.000	0.000	0.002	0.000	0.000	0.000	0.691	0.390	0.065	0.000	0.002	0.091	0.000	0.063
**3. Touch and hold**	0.000	0.000	-	0.000	0.000	0.019	0.000	0.085	0.000	0.000	0.062	0.000	0.000	0.000	0.000	0.000	0.000	0.000	0.306	0.000
**4. Rotate**	0.000	0.000	0.000	-	0.000	0.000	0.000	0.000	0.000	0.141	0.000	0.000	0.001	0.029	0.000	0.123	0.963	0.204	0.000	0.000
**5. Pinch**	0.004	0.000	0.000	0.000	-	0.000	0.050	0.000	0.131	0.000	0.000	0.007	0.191	0.016	0.774	0.000	0.000	0.002	0.000	0.850
**6. Stretch**	0.001	0.000	0.000	0.000	0.405	-	0.002	0.000	0.009	0.000	0.509	0.036	0.000	0.000	0.000	0.000	0.000	0.000	0.001	0.000
**7. Touch and slide**	0.000	0.000	0.000	0.000	0.004	0.056	-	0.000	0.954	0.000	0.000	0.351	0.001	0.000	0.190	0.000	0.000	0.000	0.000	0.125
**8. Slide**	0.000	0.000	0.000	0.000	0.000	0.000	0.000	-	0.000	0.000	0.000	0.000	0.000	0.000	0.000	0.000	0.000	0.000	0.841	0.000
**9. Rotate—2 hands**	0.000	0.000	0.000	0.105	0.000	0.000	0.000	0.000	-	0.000	0.001	0.420	0.010	0.000	0.286	0.000	0.000	0.000	0.000	0.220
**10. Press and drag—2 hands**	0.000	0.000	0.000	0.000	0.000	0.000	0.000	0.000	0.000	-	0.000	0.000	0.000	0.000	0.000	0.821	0.196	0.003	0.000	0.000
**11. Separate—2 hands**	0.018	0.000	0.000	0.000	0.161	0.253	0.010	0.000	0.000	0.000	-	0.005	0.000	0.000	0.000	0.000	0.000	0.000	0.005	0.000
**12. Join—2 hands**	0.010	0.000	0.000	0.000	0.055	0.165	0.000	0.000	0.000	0.000	0.313	-	0.000	0.000	0.047	0.000	0.000	0.000	0.000	0.025
**13. Close fist**	0.000	0.000	0.000	0.000	0.000	0.000	0.000	0.000	0.000	0.000	0.000	0.000	-	0.258	0.164	0.000	0.002	0.059	0.000	0.172
**14. Spread fingers**	0.000	0.000	0.000	0.000	0.000	0.000	0.000	0.000	0.000	0.000	0.001	0.003	0.546	-	0.019	0.000	0.038	0.420	0.000	0.018
**15. Stop with hand**	0.000	0.000	0.000	0.000	0.001	0.002	0.000	0.000	0.000	0.000	0.001	0.000	0.000	0.000	-	0.000	0.000	0.003	0.000	0.918
**16. Move with hand**	0.000	0.000	0.000	0.000	0.022	0.041	0.021	0.000	0.000	0.000	0.016	0.000	0.000	0.000	0.000	-	0.168	0.004	0.000	0.000
**17. Rotate with hand**	0.000	0.000	0.000	0.000	0.221	0.247	0.441	0.000	0.000	0.000	0.007	0.000	0.000	0.000	0.000	0.523	-	0.218	0.000	0.000
**18. Spread using both hands**	0.000	0.000	0.000	0.000	0.000	0.000	0.000	0.000	0.000	0.000	0.000	0.000	0.000	0.000	0.000	0.000	0.000	-	0.000	0.003
**19. Join using both hands**	0.000	0.000	0.000	0.000	0.001	0.009	0.000	0.000	0.000	0.000	0.060	0.122	0.529	0.897	0.000	0.000	0.000	0.000	-	0.000
**20. Rotate using both hands**	0.004	0.000	0.000	0.046	0.000	0.000	0.000	0.001	0.136	0.000	0.000	0.000	0.000	0.000	0.000	0.000	0.000	0.000	0.004	-

## Appendix B

**Table A2:** Guide Touch Screen gestures recommendation, to apply in User Interface Design for all.

Type of Gesture	Name/ How Easy to Do	Function	Gestures	Recommendations	Type of Gesture
Touch gestures using fingers on one hand	Tap	Select object		Individuals only encounter problems when the object is very small (less than 10 mm).	Ensure objects are 10 mm or larger.
	Doble tap	Select object		Individuals perform the gesture too slowly and it is recognised as two separate tap gestures rather than a double tap gesture.	Do not use; we recommend using the tap gesture.
	Touch and hold	Select and move object		This gesture is very easy to perform when being used to perform the function ‘select object’ However, this gesture proves more complicated when it is used to perform the function ‘stop object’. Individuals tend to get distracted easy and lift their finger off the screen before completing the gesture.	We recommend using this gesture for selection, although the tap gesture is more effective. Do not use for moving objects, the slide gesture would be more suitable.
	Rotate	Rotate object		Despite it being easier to perform the gesture in an anticlockwise direction, individuals rotate in a clockwise direction.	We recommend two-handed rotation gestures. Indicate direction of rotation using arrows or visual cues.
	Pinch	Shrink object		Individuals experience problems with coordination, especially with small objects.	We recommend zooming in using two fingers. If used, we recommend using this gesture to confirm critical tasks.
	Stretch	Expand object		Individuals experience problems with coordination, especially with small objects.	We recommend zooming in using two fingers. If used, we recommend using this gesture to confirm critical tasks.
	Touch and slide	Move object		Individuals tend to stop touching the screen before the gesture is finished.	Include a very visual goal and provide audible feedback to indicate when a gesture is successfully.
	Slide	Select and move object		Individuals only encounter problems when the object is very small (less than 8 mm).	Use with objects measuring 15 mm or more.
Touch gestures using fingers on both hands	Rotate	Rotate object		Despite it being easier to perform the gesture in an anticlockwise direction, individuals rotate in a clockwise direction.	We recommend two-handed rotation gestures. Indicate direction of rotation using arrows or visual cues.
	Press and drag	Move object		Individuals have difficulty coordinating fingers. They stop pressing before they finish dragging the object.	We recommend one finger.
	Separate	Expand and move object		Individuals may accidently lean on the screen with the palm of the hand.	When using this gesture makes sure other parts of the screen are no active.
	Join	Shrink and move object		Individuals may accidently lean on the screen with the palm of the hand.	When using this gesture makes sure other parts of the screen are no active.
One-handed gestures	Close fist	Shrink object		The gesture poses significant challenges. It is not detected because not all fingers are used.	Not recommended. Do not use this gesture. It is better to use the fingers on both hands.
	Spread fingers	Expand object		The gesture poses significant challenges. It is not detected because not all fingers are used.	Not recommended. Do not use this gesture. It is better to use the fingers on both hands.
	Stop with hand	Select and move object		This gesture is complicated to perform using touchscreen, but one of the easiest to perform using 3D technology.	The tap gesture is more suitable for selecting objects. The slide gesture is better for moving objects.
	Move with hand	Move object		Individuals may need to perform the gesture in several moves.	We recommend the Slide gesture. If used, allow gesture to be performed in several moves.
	Rotate	Rotate object		Individuals overcomplicate the gesture: despite it being easier to perform the gesture in an anticlockwise direction, they always rotate in a clockwise direction.	We recommend two-handed rotation gestures. Indicate direction of rotation using arrows or visual cues. If used, allow gesture to be performed in several moves.
Two-handed gestures	Spread using both hands	Move object		Individuals have a tendency to perform the gesture in several moves.	Allow gesture to be performed in several moves.
	Join using both hands	Move object		Individuals have a tendency to perform the gesture in several moves.	Allow gesture to be performed in several moves.
	Rotate using both hands	Rotate object		Although it takes several moves, it is the easiest way to rotate an object.	Recommended gesture for rotation. Allow gesture to be performed in several moves.

**Design considerations, building on those proposed by Mendoza and collaborators [60]:**	
- Use short verbal instructions - Do not use double negatives or complex words - Never give instructions in which the individual must imagine being in a different setting - Favour gestures related to the context - Limit colours to the greatest extent possible	- Use simple images that are not overly realistic - Avoid abstract images - Avoid ambiguity - Avoid using text inside images - Use a white background whenever possible - Use the most conventional form for objects
